# Intestinal schistosomiasis of Ijinga Island, north-western Tanzania: prevalence, intensity of infection, hepatosplenic morbidities and their associated factors

**DOI:** 10.1186/s12879-019-4451-z

**Published:** 2019-10-07

**Authors:** Andreas Mueller, Antje Fuss, Uwe Ziegler, Godfrey M. Kaatano, Humphrey D. Mazigo

**Affiliations:** 1 Department of Tropical Medicine, Klinikum Wuerzburg Mitte gGmbH, Medical Mission Hospital, Salvatorstrasse 7, 97074 Wuerzburg, Germany; 2 Medical Mission Institute, Salvatorstrasse 7, 97074 Wuerzburg, Germany; 3 National Institute for Medical Research, P.O. Box 1462, Mwanza, Tanzania; 4 School of Medicine, Department of Medical Parasitology, P.O. Box 1464, Mwanza, Tanzania

**Keywords:** *Schistosoma mansoni*, Prevalence, Hepatosplenic, Ijinga, Tanzania

## Abstract

**Background:**

Intestinal schistosomiasis is highly endemic in Tanzania and mass drug administration (MDA) using praziquantel is the mainstay of the control program. However, the MDA program covers only school aged children and does not include neither adult individuals nor other public health measures. The Ijinga schistosomiasis project examines the impact of an intensified treatment protocol with praziquantel MDA in combination with additional public health interventions. It aims to investigate the feasibility of eliminating intestinal schistosomiasis in a highly endemic African setting using an integrated community-based approach. In preparation of this project, we report about baseline data on *S.mansoni* prevalence, intensity of infection, related hepatosplenic morbidities and their associated factors.

**Methods:**

A cross sectional study was conducted among 930 individuals aged 1–95 years living at Ijinga Island, north-western Tanzania in September 2016. Single stool and urine samples were collected from each study participant and processed using Kato Katz (KK) technique and point-of-care Circulating Cathodic (POC-CCA) antigen test for detection of *S.mansoni* eggs and antigen respectively. Ultrasonographical examination for *S.mansoni* hepatosplenic morbidities was done to all participants. For statistical analyses Fisher’s exact test, chi-square test, student-t-test, ANOVA and linear regression were used where applicable.

**Results:**

Overall based on KK technique and POC-CCA test, 68.9% (95%CI: 65.8–71.8) and 94.5% (95%CI: 92.8–95.8) were infected with *S.mansoni*. The overall geometrical mean eggs per gram (GMepg) of faeces was 85.7epg (95%CI: 77.5–94.8). A total of 27.1, 31.2 and 51.9% of the study participants had periportal fibrosis (PPF-grade C-F), splenomegaly and hepatomegaly. Risk factors for PPF were being male (aRR = 1.08, 95%CI: 1.02–1.16, *P* < 0.01), belong to the age group 16–25 years (aRR = 1.23, 95%CI: 105–1.44, *P* < 0.01), 26–35 years (aRR = 1.42, 95%CI: 1.21–1.67, *P* < 0.001), 36–45 years (aRR = 1.56, 95%CI:1.31–1.84, *P* < 0.001) and ≥ 46 years (aRR = 1.64, 95%CI:1.41–1.92, *P* < 0.001). The length of the left liver lobe was associated with being female (*P* < 0.03), belong to the age group 1–5 years (*P* < 0.013), 6–15 years (*P* < 0.04) and *S.mansoni* intensity of infection (*P* < 0.034). Male sex (aRR = 1.15, 95%CI:1.06–1.24, *P* < 0.001) and belonging to the age groups 16–25 years (aRR = 1.27, 95%CI:1.05–1.54, *P* < 0.02) or 26–35 years (aRR = 1.32, 95%CI:108–1.61, *P* < 0.01) were associated with splenomegaly.

**Conclusion:**

*Schistosoma mansoni* infection and its related morbidities (hepatomegaly, splenomegaly, periportal fibrosis) are common in the study area. Age, sex and intensity of infection were associated with periportal fibrosis. The prevalence of *S.mansoni* was above 50% in each age group and based on the observed prevalence, we recommend MDA to the entire community.

## Background

Schistosomiasis is a neglected tropical disease which is highly prevalent in sub-Saharan Africa. An estimated 93% of the approximated 290 million people affected by the disease worldwide are living in the region [1–3]. Approximately, 120 million people have schistosomiasis related symptoms and the disease accounts for over 2.8 million years lived with disabilities [2]. In the region of sub-Saharan Africa, schistosomiasis is due to *Schistosoma mansoni* causing intestinal schistosomiasis and *S. haematobium* causing urogenital schistosomiasis [4]. Intestinal schistosomiasis caused by *Schistosoma mansoni* remains an important public health concern in Sub-Saharan Africa and is associated with significant morbidities [3–5]. Chronic *S. mansoni* infection is associated with hepatosplenic disease characterized by hepatomegaly, splenomegaly and progressive periportal fibrosis which can lead to portal hypertension, esophageal varices, liver surfaces irregularities, portal-systemic venous shunts and haemetemesis [3, 6, 7]. Many of these *S. mansoni* related morbidities can be detected and measured by ultrasonography and classified according to the World Health Organization grade scales using the Niamey protocol [8, 9].

Available evidence indicates that an estimated 8.5 million cases of chronic hepatosplenic schistosomiasis are attributed to *S. mansoni* infection in sub-Saharan Africa [3, 4]. The underlying pathophysiology is the immunological response to *S. mansoni* eggs which are carried to the liver by the blood stream. The eggs are trapped in the liver tissue around branches of the portal vein and provoke an inflammatory response mediated by CD4^+^ T-lymphocytes. Finally, this results in small fibrotic scars and over time it leads to periportal fibrosis. This process is considered to be the main pathogenetic process of portal hypertension and other hepatosplenic complications of schistosomiasis due to *S. mansoni* [7, 10]. In contrast the habitat of adult *S. haematobium* are the venous plexus of the urogenital organs which do not drain into the portal vein. *S. haematobium* is not associated with periportal fibrosis therefore. It is worth noting that epidemiological and demographic factors such as duration of residence in endemic areas [11, 12], involving in fishing activities [12], parasitological factors (low versus heavy infection intensities) [13], age (children versus adults) [5] and genetic factors [14] can contribute to development of hepatosplenic disease in endemic populations.

The World Health Organization data indicate that after Nigeria, Tanzania has the highest number of schistosomiasis cases in sub Saharan Africa [15]. In 2012 it was estimated that about 52% of the Tanzanian population (23 million people) are infected with schistosomiasis [15, 16]. The projected population of Tanzania currently stands at 55 million people [17]. In Tanzania, schistosomiasis is caused by *S. mansoni* and *S. haematobium* [5]. *Schistosoma mansoni* is focally distributed and commonly found along the large water bodies such as the Lake Victoria [5]. Indeed, multiple studies indicate, that the species *S. mansoni* is the by far predominant cause of schistosomiasis along the shoreline of Lake Victoria and on its islands [5, 12, 13, 16, 18, 19, 20, 21, 22]. Communities living there are disproportionally highly affected by the *S. mansoni*-specific pathology and a number of studies have reported a high prevalence of hepatosplenic morbidities in this region [5, 12, 13, 23, 18]. A study among 360 school children on Ukerewe Island in Lake Vicctoria found splenomegaly in 90,7%, right liver lobe hepatomegaly in 89% and overt signs of periportal fibrosis in 5,4% of the participants [5]. The disease is highly common among school children and adults [19, 24], of recent, data have emerged showing that pre-school aged children are also infected and carry heavy infection intensities [20].

At present, the main control approach against intestinal schistosomiasis in Tanzania focuses on mass drug administration (MDA) with praziquantel [5]. The MDA approach mainly targets school aged children (SAC) or children attending primary schools and drugs are offered at the school environment [25]. This approach has been advocated as very cost-effective, especially when drugs are distributed irrespective of the infection status. Repeated rounds of MDA have been reported to result in reduction of prevalence of infection, intensity of infection and reversibility of enlarged organs [25, 26, 27]. It should be noted that despite the implementation of several rounds of annual MDA in Tanzania, the prevalence of the disease remains very high in many areas surrounding Lake Victoria [5, 19, 20, 21]. It is unlikely that a control strategy based on MDA alone will result into the interruption of transmission and elimination of the disease in high prevalence areas. Understanding this, World Health Assembly (WHA) in its 2012 resolution 65.21 [14] called for integrated control approaches which are mainly characterised by inclusion of public health education, access to clean water, improved sanitation and snail control into the MDA program [24]. Following this concept, we launched a pilot study on Ijinga island in Lake Victoria where we combine an intensified MDA program with repeated health education and WASH interventions. In preparation of this 5-year project, we collected baseline data which will be useful for monitoring the impact of the implemented intervention measures at the study area. Thus, the present study reports data on *S. mansoni* prevalence, intensity of infection, related hepatosplenic morbidities and their associated factors.

## Methods

### Study area

The present study was conducted at Magu district, specifically on Ijinga Island located on the southern shore of the Lake Victoria in north-western Tanzania. Ijinga Island and Magu district are located at 2° South of the equator and 33°-34° East. The area is characterized by a tropical type of climate with bimodal rain seasons, short rain season running from October to December and long rain seasons starting from March to May. The average annual temperature is 26.5 °C, average annual rainfall is 1200 mm [28]. The island has a total of 420 households and is inhabited by a total of 2520 permanent residents. The majority of the inhabitants are involved in subsistence farming, fishing and livestock keeping. In the area the main source of water for domestic and recreational use for the inhabitants is Lake Victoria. Because of the high water contact levels, inhabitants have a high risk of becoming infected with *S. mansoni* and the high exposure maintains high intensities of the infection into adulthood. Annual mass drug administration (MDA) using praziquantel is mainly conducted among school children within the school environment, other community members are not involved, especially adult and non-school going children (pre-school children and other age groups out of the school).

### Study design, sample size, inclusion and exclusion criteria

The study was designed as analytical cross-sectional survey conducted between September and October 2016 on Ijinga Island. The study included all individuals aged from 1 year, who were permanent inhabitants of the study area as per our house to house census and gave consent/assent for participation in the study. The sample size was determined using a formula for binary outcome (*S. mansoni* infection) in a cross-sectional study design, developed by Leslie Kish [29]: n = z^2^p(1-p)/d^2^ (n = sample size, z = level of confidence, p = expected prevalence in proportion of one, d = precision in proportion of one). For the conventional level of confidence of 95% the Z value was 1.96. Considering the prevalence of *S. mansoni* of 47.8% [23], in four villages along the shore of Lake Victoria, at 95% confidence interval and margin errors of 3.2%, the minimum sample size required was 936. Considering 10% of refusal and non-responding (addition of 94 participants), a total sample size of 1030 was required for this study. A few days prior to the study a team of community health care workers visited all households for individual counselling and invited all permanent residents to take part. Participation was voluntarily and entirely free of charge. All inhabitants of the island who wished to be examined and did not receive Praziquantel during the last 3 months were eligible for participation. The investigations were carried out during an entire week including the weekend allowing also the working population to take part in the study and to minimize a sampling bias. A total of 930 participants participated voluntarily in this present study. Despite the fact that the sample size was below the minimum sample size, the power of the study remained above 90%.

### Data collection

(i) Interview using questionnaire

A pre-tested questionnaire was used to collect demographic information from study participants (age, sex, and residence). The history of participations in the mass drug administration program was recorded from school aged children, adolescents and adults. For adult participants additional questions on economic activities were included.

(ii) Parasitological examination of *Schistosoma mansoni* eggs using Kato Katz technique

From each study participant, a single stool sample was collected using a labelled stool container and from each collected sample two Kato Katz thick smears were prepared using the Kato Katz technique with a template of 41.7 mg per thick smear [30]. Two experienced laboratory technicians examined all the Kato Katz thick smears at the National Institute for Medical Research, Mwanza, Tanzania. For quality assurance, 15% of all positive and negative Kato Katz slides were re-examined by a third independent laboratory technician who was blinded of the results of the first two laboratory technicians.

(iii) Examination of *Schistosoma mansoni* Circulating Cathodic Antigen (CCA) test in urine samples

A point-of-care Circulating Cathodic Antigen (CCA) test (Rapid Medical Diagnostic- http://www.rapid-diagnostics.com/, batch number 50182) was used to screen for CCA antigen in urine samples. A single urine sample was collected from each of the study participants and examined for CCA antigen based on the manufacturer’s instructions (Rapid Medical Diagnostic- http://www.rapid-diagnostics.com/). Trace results of the test were considered as positive. Laboratory technicians trained on POC-CCA test and with experience from previous studies performed the test. The entire laboratory team involved in CCA examination was blinded of the Kato Katz results.

(iv) Ultrasound examinations of *Schistosoma mansoni* related morbidities

All study participants were clinically examined for consistency of the liver and the spleen using the approach described elsewhere [31]. Three ultrasonographers (medical doctors with long experience of ultrasound examination) participated in the ultrasonographical examination of the study participants. For quality assurance, 10% of the study participants were re-examined by all three ultrasonographers at different points and the results of the participants were agreed between them. In case of any inconsistence/disagreement, participants were requested to be re-examined. Classification of the ultrasound detected *S. mansoni* related morbidities was done using the modified Niamey Protocol [8]. The spleen size, peripheral portal branches (PPBs), periportal fibrosis, liver texture patterns, thickness of the peripheral portal branches were classified based on the Niamey protocol [8]. The degree of periportal fibrosis was qualitatively classified based on the liver image patterns categorized as A, B, C, D, E and F as per Niamey protocol [8, 17]. The liver image/texture patterns A and B were classified as normal.

The size of the left liver lobe was measured along the right para-sternal line (PLL). For the spleen size, the longest diameter was taken along the craniocaudal axis of the spleen from the left flank side. The portal vein diameter (PVD) was measured at a midway between the confluence of the splenic vein and the superior mesenteric vein and its bifurcation inside the liver. The length of the left liver lobe, spleen and the diameter of the portal vein were adjusted using the height data from the reference population [8].

### Data analysis

All collected data were double entered into an excel sheet, cleaned and imported to Stata Version 15 (StataCopr, 2017, Statistical software, College Station, TX: StataCorp LP. Texas, USA). All categorical variables were summarized using percentages and comparison of categorical/proportions variables either using Fisher’s exact or chi-square tests (χ^2^). All continuous variables were analyzed using univariate test and reported as means or median with their standard deviation or interquartile ranges depending on the nature of distributions of the continuous variables. The Geometrical mean eggs per gram of feaces (GMepg) for *S. mansoni* eggs were calculated for each study participant from the non-logarithmically transformed means. Comparison of mean eggs count for *S. mansoni* between different demographic characteristics of the study participants was done using student-t-test (two groups) or ANOVA (more than two groups). Intensity of infection was categorized as 1–99 epg (low), 100–399 epg (moderate), ≥400epg (heavy) intensities [32]. The ultrasound height adjusted data for the liver, spleen and portal vein diameter were compared between the infected and uninfected participants using the t-test. The Niamey protocol was used to define the cut-off point between normal and enlarged spleen, left liver lobe and dilatation of the portal vein dilatation [8].

Generalized linear model (glm) and the linear regression model were used to assess the contributions of intensities of *S. mansoni*, age, sex and infection status on the extent of the liver and spleen enlargements. The glm was used to determine risk factors associated with periportal fibrosis and splenomegaly while the linear regression model was used to assess predictors of increased height of the left liver lobe. Linear regression model was constructed for height-adjusted measurements of the left liver lobe and the above mentioned explanatory variables were included. At bivariate analysis, explanatory variables with *P*-value < 0.2 were considered for multivariable analyses. For the glm, Risk Ratios with their 95%CI were generated and used to measure the level of risk and the Beta coefficients with their corresponding 95%CI were also generated from the linear regression. The *P*-values of < 0.05 were considered significant.

### Treatment

All participants who were diagnosed with *S. mansoni* infection were treated with a single dose of praziquantel (40 mg/kgBwt). Treatment was done under direct observation (DOT) of qualified nurses. All study participants received food before taking the drug to minimize possible side effects of praziquantel. After taking drugs, participants were under direct observation for two hours before leaving the treatment area and asked to report any side effects to the study team. In case of abdominal pain, symptomatic treatment with paracetamol and n-butylscopolamin was used. Children aged < 6 years received crushed tablets mixed with juice according to their body weight.

## Results

### Characteristics of the study participants

A total of 930 participants were enrolled in the present study, of these 513 (55.2%) and 417 (44.8%) were female and male respectively. The mean age of the study participants was 23.3 ± 18.5 years. Of the study participants 87.2% reported to have never participated in a mass drug administration campaign against schistosomiasis. Only 12.8% reported to have participated at least once in the program. Table 1 shows the age and sex distribution of the study participants. All participants were from Ijinga Island, the majority of the adult participants reported to be involved in subsistence farming, livestock keeping and fishing.

**Table 1:** Age and sex distribution of the study participants on Ijinga Island, north-western Tanzania

Sex	Age groups (in years)					
	1–5	6–15	16–25	26–35	36–45	≥ 46
Female	45 (59.2%)	213 (50.9%)	65 (54.6%)	55 (53.4%)	65 (71.4%)	73 (57%)
Male	29 (40.8%)	205 (49%)	54 (45.4%)	48 (46.6%)	26 (28.6%)	55 (42.9%)
Total	71	418	119	103	91	128

### Prevalence and intensity of infection of *Schistosoma mansoni* using Kato Katz technique

The overall prevalence of *S. mansoni* was 68.9% (95%CI: 65.8–71.8). The prevalence of *S. mansoni* among male individuals was 74.3% (95%CI: 69.9–78.3) and 64.5% (95%CI: 60.3–68.6) in female participants. This gender difference was significant (χ^2^ = 10.3523, *P* < 0.001). In the age group of 6–15 years the prevalence of *S. mansoni* was 86.1% (95% CI: 82.4–89.1) and among the 16–25 years old the prevalence was 57.1% (95%CI: 47.9–65.8). Compared to the other age groups these 2 age groups of SAC, adolescents and young adults had a significantly higher prevalence of *S. mansoni* (χ^2^ = 105.61, *P* < 0.001). Table 2 shows the prevalence of *S. mansoni* categorized by sex and age.

**Table 2:** Prevalence of *Schistosoma mansoni* categorized by age and sex

Variable		Number examined	Prevalence (%, n)	95% CI	χ^2^	*P*-value
Sex	Female	513	64.5% (331)	60.3–68.6	10.3523	0.001
	Male	417	74.3% (310)	69.9–78.3		
Age (in years)	1–5	71	54.9% (39)	43.0–66.3	105.61	0.001
	6–15	418	86.1% (360)	82.5–89.1		
	16–25	119	57.1% (68)	47.9–65.8		
	26–35	103	55.3% (57)	45.4–64.7		
	36–45	91	51.7% (47)	41.3–61.8		
	≥46	128	54.7% (170)	45.8–63.2		

The overall geometrical mean eggs per gram of faeces (GMepg) was 85.7epg (95%CI: 77.5–94.8), with no sex differences (89.9GMepg for male versus 81.4GMepg for female, t = − 0.2887, *P* = 0.77). There was a significant difference in GMepg between the age groups 1–5 years (91,9GMepg, 95% CI:58.8–143.5) and 6–15 years (108.6GMepg, 95% CI:95.2–124.0) (F-test = 10.71, *P* < 0.0001). The age group 6–15 years had the highest intensity of infection among all age groups. Table 3 shows the Geometrical mean eggs per gram of faeces of *S. mansoni* stratified by sex and age. Based on the WHO classification of intensity of infection, majority of the study participants had light (55.2%) to moderate (20.4%) intensity of infection. Only 12.9% of them had a heavy intensity of infection.

**Table 3:** Geometrical mean eggs per gram of faeces of *S. mansoni* stratified by age and sex

Variables		GMepg	95%CI	T-test or F-test
Sex	Female	89.9	78.1–103.7	t-test = −0.2887, *P* = 0.77
	Male	81.4	70.4–94.0	
Age groups (in years)	1–5	91.9	58.8–143.5	F-test = 10.71, *P* < 0.0001
	6–15	108.6	95.2–124.0	
	16–25	88.1	65.0–119.3	
	26–35	61.7	43.2–88.1	
	36–45	42.8	30.8–59.8	
	≥ 46	49.3	37.3–65.0	

### Prevalence of *Schistosoma mansoni* based on circulating cathodic antigen tests

The laboratory results for the point-of-care Circulating Cathodic Antigen (POC-CCA) tests were available for 929 study participants. The overall prevalence of *S. mansoni* based on the POC-CCA test was 94.5% (95%CI: 92.8–95.8). The male individuals recorded the highest prevalence of *S. mansoni* compared to female individuals (96.2% versus 93.2%, χ^2^ = 3.9839, *P* = 0.046). In relation to age groups, the age groups 6–15 years recorded the highest prevalence of infection compared to other age groups (98.1%, χ^2^ = 34.2723, *P* < 0.001). Table 4 below shows the prevalence of *S. mansoni* infection based on POC-CCA test stratified by age and sex.

**Table 4:** Prevalence of *Schistosoma mansoni* based on point-of-care Circulating Cathodic Antigen (POC-CCA) test stratified by age and sex

Variables		No. examined	Prevalence (%, n)	95%CI	χ^2^	*P*-value
Sex	Female	512	93.2%(477)	90.6–95.1	3.9839	0.046
	Male	417	96.2%(401)	93.8–97.6		
Age (in years)	1–5	71	95.8%(401)	87.4–98.7	34.2723	0.001
	6–15	418	98.1%(410)	96.2–99.0		
	16–25	119	91.6%(109)	84.9–95.5		
	26–35	103	88.4%(91)	80.4–93.3		
	36–45	91	97.8%(89)	91.4–99.5		
	≥ 46	127	87.4%(111)	80.3–92.2		

### Prevalence of periportal fibrosis and associated factors

The overall prevalence of definite periportal fibrosis (PPF) (grade C-F) was 27.1% (95%CI: 24.3–30.1). 58,2% of the participants showed a normal liver pattern (grade A), 14,7% a grade B pattern, which was considered as not distinct for PPF according to Kaatano et al. [33] and King et al. [34]. In relation to sex of the study participants, the prevalence of PPF was higher in male individuals than in female individuals (*P* < 0.026). In general, the oldest age groups, 36–45 years and ≥ 46 years had a higher prevalence of PPF compared to other age groups (*P* < 0.001). In relation to infection status, the findings indicate that individuals who had no *S. mansoni* detectable eggs in their stool samples recorded the highest prevalence of PPF (*P* < 0.005). The findings indicated that there was no significant difference in PPF prevalence in relation to intensity of infection categories (*P* = 0.42). Table 5 shows the prevalence of periportal fibrosis in relation to demographic characteristics of the study participants, infection status and intensity of infection.

**Table 5:** Prevalence of periportal fibrosis in relation to demographic characteristics of the study participants, infection status and intensity of infection

Variable		No. examined	PPF (%,n)	95%CI	χ^2^	*P*-value
Sex	Female	513	24.2% (124)	20.7–28.1	4.9559	0.026
	Male	417	30.7% (128	26.4–35.3		
Age (in years)	1–5	71	8.5% (6)	3.7–17.8	131.0316	0.001
	6–15	418	13.2% (55)	10.2–16.8		
	16–25	119	31.1% (37)	23.3–40.1		
	26–35	103	39.8% (41)	30.6–49.6		
	36–45	91	47.3% (43)	37.1–57.7		
	≥46	128	54.7% (70)	45.8–63.2		
*S. mansoni* infection status	Infected	641	24.3% (156)	21.2–27.8	7.9531	0.005
	Not infected	289	33.2% (96)	27.9–38.8		
Intensity of infection	1–99	354	25.9% (92)	21.7–30.8	1.7427	0.42
	100–399	204	21.1% (43)	15.9–27.3		
	≥400	83	25.3% (21)	16.9–35.9		

At bivariate analysis, being a male, belonging to age groups 16–25 years, 26–35 years, 36–45 years and ≥ 46 years were associated with periportal fibrosis (Table 6). On multivariable analysis the following variables were associated with periportal fibrosis **(**Table 6):

- being male (aRR = 1.08, 95%CI: 1.02–1.16, *P* < 0.01),
- belonging to the age group 16–25 years (aRR = 1.23, 95%CI: 1.05–1.44, *P* < 0.01),
- belonging to the age group 26–35 years (aRR = 1.42, 95%CI: 1.21–1.67, *P* < 0.001),
- belonging to the age group 36–45 years (aRR = 1.56, 95%CI: 1.31–1.84, *P* < 0.001)
- and belonging to the age group ≥46 years (aRR = 1.64, 95%CI: 1.41–1.92, *P* < 0.001)

**Table 6:** Risk factors associated with periportal fibrosis on Ijinga Island, north-western Tanzania

Variable	RR	95%CI	*P*-value	aRR	95%CI	*P*-value
Sex						
Female	1			1		
Male	1.07	1.01–1.13	0.03	1.08	1.02–1.16	0.01
Age groups (in years)						
1–5	1			1		
6–15	1.05	0.94–1.16	0.38	1.06	0.92–1.21	0.39
16–25	1.25	1.11–1.42	0.001	1.23	1.05–1.44	0.01
26–35	1.36	1.21–1.55	0.001	1.42	1.21–1.67	0.001
36–45	1.47	1.29–1.68	0.001	1.56	1.31–1.84	0.001
≥ 46	1.59	1.41–1.79	0.001	1.64	1.41–1.92	0.001
Infection status						
Uninfected	1					
Infected	0.91	0.86–0.97	0.005	–	–	–
Intensity of infection (epg)						
1–99	1			1		
100–399	0.95	0.88–1.03	0.19	1.00	0.94–1.08	0.92
≥ 400	0.99	0.89–1.10	0.89	1.12	1.01–1.23	0.02

### Left liver lobe hepatomegaly, splenomegaly and their associated factors or predictors

Data on left liver lobe measurements were available for 909 study participants. Overall, 51.9% (472/909, 95%CI: 48.7–55.2) of the study participants had enlarged and grossly enlarged left liver lobe. Of these, 79% (373/472) and 21% (99/472) had enlarged and grossly enlarged left liver lobe respectively. In relation to sex, female study participants had higher prevalence of enlarged (59.5% versus 40.5%) and grossly enlarged left liver lobe (62.6% versus 37.4%, χ^2^ = 10.0110, *P* < 0.01). In the age groups, the age group 6–15 years recorded the highest prevalence of enlarged (49.6%) and grossly enlarged (36.4%) left liver lobe compared to other age groups (χ^2^ = 29.1165, *P* < 0.001). No statistical difference in prevalence of enlarged and grossly enlarged left liver lobe was observed in relation to *S. mansoni* infection status (*P* = 0.82) and intensity of infection categories (*P* = 0.47).

Splenomegaly was detected in 31.2% (278/892, 95%CI: 28.2–34.3) of the study participants whose data were available. Of these participants, 212/278 (76.3%) and 66/278 (23.7%) had moderately to marked enlarged splenomegaly. Male individuals recorded a higher prevalence of moderately (27.8%) and markedly enlarged spleen (9.5%) than the female individuals (χ^2^ = 13.3959, *P* < 0.001). Similarly, there was a significance difference in prevalence of moderately and markedly enlarged spleen in relation to *S. mansoni* infection status. Individuals who had *S. mansoni* detectable eggs in their stool samples had a higher prevalence of moderately (26.1%) and markedly enlarged spleen (7.7%) (χ^2^ = 6.6121, *P* < 0.037).

Hepatosplenomegaly (enlargement of both spleen and liver) was detected in 55.8% (155/895) of the study participants. Other morbidities detected were ascites (0.2%), collateral veins (1.7%) and gall bladder wall thickening (9.7%).

On linear regression model using height adjusted ultrasound measurements of the left liver lobe as a continuous outcome variable for individuals who had detectable *S. mansoni* eggs in stool samples, the findings demonstrated that, the ultrasound adjusted left liver lobe measurements were significantly associated with being female (*P* < 0.03), being in the age groups 1–5 years (*P* < 0.013), 6–15 years (*P* < 0.04) and *S. mansoni* intensity of infection (*P* < 0.034) (Table 7).

**Table 7:** Predictors of height adjusted left liver lobe size among residents of Ijinga Island infected with *S. mansoni*

Variables	β-coefficient	SE	95%CI	*P*-value
Sex				
Female	1			
Male	0.24	0.1080857	0.03–0.45	0.03
Age groups (in years)				
1–5	0.8876	0.2503498	0.39–1.38	0.001
6–15	0.3797	0.18022	0.03–0.733	0.035
16–25	−0.1455	0.2199547	−0.58-0.28	0.51
26–35	1			
36–45	0.5855402	0.2361574	0.12–1.05	0.013
≥ 46	0.305517	0.2153395	−0.12-0.72	0.16
*Schistosoma mansoni* intensity (epg)				
*S.mansoni* intensities	0.004317	0.0002037	0.00003–0.008316	0.034

R^2^ = 0.0313 (adjusted R-square), F = 5.20, *P* < 0.0001

Linear regression model was constructed for height adjusted ultrasound measurements of the left liver lobe (organ size as continuous variable, i.e. deviations from mean)

On bivariate analysis, factors associated with splenomegaly were mainly being male, belong to the age groups 16–25 years and 26–35 years and having detectable *S. mansoni* eggs in stool samples. Similarly, in the multivariable analysis, being male (aRR = 1.15, 95%CI: 1.06–1.24, *P* < 0.001), belong to the age group 16–25 years (aRR = 1.27, 95%CI: 1.05–1.54, *P* < 0.02) and age group 26–35 years (aRR = 1.32, 95%CI: 1.08–1.61, *P* < 0.01) (Table 8).

**Table 8:** Factors associated with splenomegaly

Variables	RR	95%CI	*P*-value	aRR	95%CI	*P*-value
Sex						
Female	1					
Male	1.12	1.05–1.88	0.001	1.15	1.06–1.24	0.001
Age groups (in years)						
1–5	1			1		
6–15	1.11	0.97–1.25	0.10	1.07	0.95–1.21	0.28
16–25	1.19	1.03–1.37	0.02	1.18	1.03–1.36	0.02
26–35	1.16	1.01–1.35	0.04	1.16	1.01–1.34	0.04
36–45	1.09	0.93–1.26	0.27	1.10	0.95–1.28	0.19
≥ 46	1.02	0.89–1.18	0.73	1.02	0.89–1.17	0.74
Infection status						
Uninfected	1			1		
Infected	1.08	1.02–1.16	0.013	1.08	1.01–1.16	0.02
Intensity of infection (epg)						
1–99	1					
100–399	1.00	0.92–1.09	0.98	–	–	–
≥ 400	1.00	0.88–1.12	0.99	–	–	–

### Comparison of the height adjusted mean deviations of organs in relation to infection with *S. mansoni*

The mean deviations of ultrasound measurements of left liver lobe, spleen and portal vein were compared between individuals who were infected or uninfected with *S. mansoni* based on Kato Katz technique. Overall, the height-adjusted ultrasound measurements of the left liver lobe (t = − 0.7280, *P* = 0.46) did not differ between the two groups. There was a significant difference in height-adjusted ultrasound measurements of the spleen (t = − 2.0616, *P* < 0.04) and portal vein (t = 6.2970, *P* < 0.001) between individuals who were infected or not with *S. mansoni*. For the height-adjusted length of the spleen, individuals who were infected showed an enlargement of the spleen compared to uninfected individuals. For the portal vein, individuals who were infected had more dilated (increased diameter) portal vein diameter compared to uninfected individuals.

## Discussion

The findings from this study indicated that Ijinga Island is highly endemic for *S. mansoni* infection. The prevalence of *S. mansoni* and its related intensity of infection is high in all age groups. Furthermore, our findings noted a difference in case detection of *S. mansoni* infection between Kato Katz (KK) technique and point-of-care Circulating Cathodic Antigen test. The POC-CCA test detected more cases of the disease than KK technique. The findings further noted that *S. mansoni* related hepatosplenic morbidities are also common in all age groups and both sexes. Heavy intensity of *S. mansoni* infection was associated with hepatomegaly and splenomegaly. A number of demographic and epidemiological factors were noted to be partly contributing to the development *S. mansoni* related hepatosplenic morbidities in the study area.

Findings on the prevalence of *S. mansoni* infection on Ijinga Island corroborate the results of previous studies which demonstrated that communities along the southern shore line of Lake Victoria and its Islands carry the highest burden of the disease [12, 13, 18, 19, 23, 35]. Our findings noted a very high prevalence using both KK technique and POC-CCA test. Based on KK technique, the prevalence of *S. mansoni* observed in this study was lower than the 78% [18] and 86.3% [13] recorded at the nearby Island of Ukerewe north-western Tanzania. Other previous studies in the same area have recorded lower prevalences [19, 23] than in the present study. A possible explanation could be the low rate of participation in previous MDA campaigns (12.8% acc. to the questionnaire) of the Ijinga community. The age and sex differences in *S. mansoni* infection status recorded in this present study were comparable to results of previous similar studies conducted in schistosomiasis endemic areas [18, 36, 37]. The variations in *S. mansoni* prevalence between age groups and sex is mainly explained by varying patterns of exposure to risk areas (water contacts) and development of acquired partial immunity [13, 36, 38]. The high prevalence of > 50% of *S. mansoni* infection in all the age groups based on KK technique and POC-CCA test indicate that Ijinga island is an old focus for *S. mansoni* transmission and for successful control of the disease and its related hepatosplenic morbidities, the entire community should receive at least two rounds of MDA per year according to WHO recommendation [39].

Of note to our findings is the high prevalence of *S. mansoni* infection in pre-school age children (≤6 years) based on both KK technique and POC-CCA test. The age group had also high intensity of *S. mansoni* infection compared to the older age group. These findings indicate that, *S. mansoni* infections starts at an early age and with heavy infection intensities, the age group is likely to start developing hepatosplenic disease at early age. At the time these children reach maturity if left untreated, are likely to end up with severe hepatosplenic disease [40]. Similar studies in East Africa have recently reported a high prevalence and intensity of infection of *S. mansoni* in the same age group using both KK technique and POC-CCA test [20, 22]. It is worthwhile noting that the use of KK technique alone in PSAC children results into underestimation of the prevalence of the infection. Therefore, it remains important to combine this technique with a more sensitive diagnostic technique such as POC-CCA test to increase the detection of true cases of the disease in this age group [20, 41, 42]. In our present study, almost 41% of the pre-school children were missed by KK technique. Taking into account that the peadiatric formulation of praziquantel will be available in the near future [40, 43], which will allow including of PSAC into the MDA program, it is important to include a more sensitive diagnostic test for case detection in this age group and for monitoring of drug efficacy [40].

The results of the present study show that ultrasound detectable hepatosplenic morbidities are common among our study population and almost exclusively are attributed to *S. mansoni* infection. Our results confirm the findings of the previous studies conducted in the same region which noted different patterns of organ morbidities in communities living along the shoreline of the lake and on islands [12, 13, 18, 21]. The common hepatosplenic morbidities detected in the present study population were periportal fibrosis (PPF), hepatomegaly, splenomegaly, hepatosplenomegaly and gall bladder wall thickening. The prevalence of PPF observed in the present study was lower than the 41% [18] and 42% [33] reported from Kome and Msozi village at Ukerewe district respectively. However, other similar studies in the same region have reported lower prevalences than what was recorded in this area [12, 21]. In the same region, hepatomegaly and splenomegaly related to *S. mansoni* infection is a common observation, for instance on Ukerewe Island, 35 and 80% of the studied population had left liver lobe hepatomegaly and splenomegaly [13]. In the nearby region of Mara, 28.5 and 29.6% of the study participants had splenomegaly and hepatomegaly [21] while on Kome Island, 68 and 55% of the adult individuals were ultrasonographically detected to have hepatomegaly and splenomegaly [33]. Authors in previous similar studies have described a number of demographic and epidemiologic factors which partly could explain the variation in prevalence of ultrasound detectable *S. mansoni* related morbidities between communities living in different transmission settings [11, 12, 36, 37].

Interestingly, our findings noted a degree of hepatosplenic morbidities among children aged < 5 years. The main hepatosplenic morbidities observed in the children was mainly left liver lobe enlargement, splenomegaly and only a small proportion of the children had signs of PPF. It should be noted that at such young age, is difficult to precisely classify the liver image pattern/grades as detected by ultrasound [22]. An intensive inflammatory response due to the accumulation of *S. mansoni* eggs in the liver tissue, especially the left liver lobe could partly explain the liver size enlargement in this age group [7, 38]. Studies have reported an association between the enlargement of the left liver lobe and *S. mansoni* infection and its intensity [11, 36, 37]. The main concern here, is the development of *S. mansoni* related hepatosplenic morbidities at such young age and the fact that, to date this age group is not considered for MDA with praziquantel [40, 43, 44]. At the time when these children will be attending primary school, they are at risk of having already developed advanced stages of hepatosplenic disease. A recent study from Uganda, has pointed out the growing problem of hepatosplenic disease among pre-school aged children living in different transmission settings for *S. mansoni* infection [22]. In this study, PSAC were noticed to have already developed liver image grades suggestive of PPF (Symmer’s pipe stems fibrosis) at six years of age [22]. This observation is commonly seen in adult population with long-standing infection with *S. mansoni* [18, 45]. Cumulatively, our observation and that of other authors in Uganda [22] on the development of hepatosplenic disease in this young age group suggest that the period from infection to the development of hepatosplenic morbidities is short compared to previous knowledge based on adult. It seems that *S. mansoni* hepatosplenic morbidities in the PSAC have been underestimated if not overlooked in many studies. It is high time now for this age group to be considered for appropriate treatment [44].

In the present study, the main risk factors associated with PPF were being a male, aged above 16 years and having heavy intensity of infection. The association of male sex with PPF mainly depicts the level of exposure and high infection intensity observed among male individuals in endemic areas [14, 18, 37]. The relationship between increased age and observation of chronic hepatosplenic morbidities in older ages mainly signifies that, *S. mansoni* is a chronic infection which requires time for the overt related morbidities such as PPF, hepatomegaly and splenomegaly to develop [11, 14, 37]. Risk factors such as village of residence, age, occupation (being fisherman), being male has been shown by previous studies to be associated with PPF in *S. mansoni* endemic areas [11, 36, 37]. Heavy intensity of *S. mansoni* infection was also noted in this present study to be associated with PPF. Similar studies have repeatedly described this observation in individuals with severe PPF grades [14, 37].

On the other hand, our findings noted a significant correlation between the heights adjusted length of the left liver lobe with *S. mansoni* intensity of infection, female individuals and age groups. Similarly, young age groups, having detectable *S. mansoni* eggs in stool sample and being male were associated with enlargement of the spleen. These explanatory factors have been previous described to interact in causing *S. mansoni* related hepatosplenic disease characterized by hepatomegaly, splenomegaly and hepatosplenomegaly in different settings [11, 36, 37]. In endemic areas, male individuals and children in young age groups are more exposed to water sources than other members of their communities and therefore accumulate heavy intensities of infection with increasing age. This partly explains the high prevalence of some of the hepatosplenic morbidities, for instance PPF and left liver lobe hepatomegaly among male individuals compared to females [14]. In the young age groups, left liver lobe hepatomegaly and splenomegaly have been noted to correlate with heavy intensities of *S. mansoni* infection [22, 36, 37]. It is worthwhile noting that, in schistosomiasis endemic areas, *P. falciparum* malaria is also a cause of hepatomegaly and splenomegaly and differential diagnosis especially in young age groups is a requirement [46].

The following limitation have to be discussed when interpretation of our results is done. The use of single stool sample for examination may have underestimated the intensity of *S. mansoni* infection taking into account the day to day variability of parasites eggs output. Also, we did not screen for malaria parasites especially in the young age groups, which could confound our findings on the enlargement of the left liver lobe and spleen.

The prevalence of Hepatitis B surface antigen positive chronic hepatitis B among the adult population of Tanzania is about 7% [47]. It has to be assumed that co-infections with *S. mansoni* are common and chronic viral hepatitis contributes to the severity of liver disease. Nevertheless, the ultrasound patterns of liver cirrhosis or mixed pathology were not detected among our study participants. A screening for HBs-antigen would have required to take a blood sample what we considered to be an obstacle for a high rate of voluntary participation.

Our findings reflect the situation of schistosomiasis in many of the communities living on the islands of Lake Victoria, north-western Tanzania and we consider the conclusions drawn as transferable.

## Conclusion

Our findings indicate that, *S. mansoni* infection and its related hepatosplenic morbidities are prevalent in the study area and the hepatosplenic morbidities show an association with with age and sex. The prevalence of *S. mansoni* infection was above 50% in all age groups and the youngest age groups had higher intensities of infection. Hepatosplenic morbidities were also detected among the children below < 5 years of age. Based on the observed prevalence of the infection in all age groups, the entire community of Ijinga Island including pre-school children needs to be targeted for mass drug administration with praziquantel. We propose two to three rounds of MDA per year to reduce the prevalence and then revise to single annual dose for the entire community.

## Data Availability

The datasets collected and/or analyzed during the current study are available from the corresponding author upon reasonable request. However, we did not receive permission to share the raw data from the institution review boards.

## Abbreviations

KK: Kato Katz technique
MDA: Mass Drug Administration
POC-CCA: Point-of-care Circulating Cathodic Antigen
PPF: Periportal fibrosis
PSCA: Pre-school aged children
SAC: School aged children
WHO: World Health Organization
